# Changes in growth, anaemia, and iron deficiency among children aged 6–23 months in two districts in Nepal that were part of the post‐pilot scale‐up of an integrated infant and young child feeding and micronutrient powder intervention

**DOI:** 10.1111/mcn.12693

**Published:** 2018-10-12

**Authors:** Lindsey M. Locks, Pradiumna Dahal, Rajkumar Pokharel, Nira Joshi, Naveen Paudyal, Ralph D. Whitehead, Stanley Chitekwe, Zuguo Mei, Bikash Lamichhane, Aashima Garg, Maria Elena Jefferds

**Affiliations:** ^1^ Department of Nutrition Harvard TH Chan School of Public Health Boston Massachusetts; ^2^ Nutrition Section United Nations Children's Fund (UNICEF) Headquarters New York New York; ^3^ Nutrition Section United Nations Children's Fund (UNICEF) Kathmandu Nepal; ^4^ Nepal Ministry of Health and Population Kathmandu Nepal; ^5^ New ERA Kathmandu Nepal; ^6^ Nutrition Branch, Division of Nutrition, Physical Activity, and Obesity United States Centers of Disease Control and Prevention Atlanta Georgia

**Keywords:** child growth, child nutrition, infant and young child feeding, micronutrient powders

## Abstract

There is limited research on integrated infant and young child feeding (IYCF) and micronutrient powders (MNPs) programmes operating at scale, despite widespread implementation. This study uses cross‐sectional baseline (*n* = 2,542) and endline (*n* = 2,578) surveys representative of children 6–23 months in two districts in Nepal that were part of a post‐pilot scale‐up of a IYCF–MNP programme. Multivariable log‐binomial regression models were used to estimate prevalence ratios (PRs) for stunting (length‐for‐age *z*‐score <−2), wasting (weight‐for‐length *z*‐score <−2), underweight (weight‐for‐age *z*‐score <−2), anaemia (altitude‐adjusted haemoglobin <110 μg/L), moderate or severe anaemia (altitude‐adjusted haemoglobin <100 g/L), iron deficiency (inflammation‐adjusted ferritin <12 μg/L), and iron deficiency anaemia (iron deficiency + anaemia [IDA]) at endline versus baseline and also to compare children in the endline survey based on frequency of mothers' interactions with female community health volunteers (FCHVs; >1× per month or monthly vs. <1× per month) and MNP coverage (1 or ≥2 distributions vs. none among children 12–23 months). Endline children were significantly less likely to be stunted than baseline children in both districts (multivariable‐adjusted PR [95% CI]: 0.77 [0.69, 0.85], *P* < 0.001 and 0.82 [0.75, 0.91], *P* < 0.001 in Kapilvastu and Achham, respectively); however, only Achham had significantly lower prevalences of underweight, moderate/severe anaemia, iron deficiency, and IDA at endline. At endline, 53.5% and 71.4% of children had tried MNP in Kapilvastu and Achham districts, respectively, consuming an average of 24 sachets from the last distribution. Frequent maternal–FCHV interactions were associated with a reduced risk of stunting and underweight at endline, whereas repeat MNP coverage was associated with reduced risk of anaemia and IDA. Future research using experimental designs should verify the potential of integrated IYCF–MNP programmes to improve children's nutritional status.

**Key messages**

- In two districts in Nepal that were part of a post‐pilot scale‐up of an integrated infant and young child feeding–micronutrient powder (IYCF–MNP) programme, the prevalence of stunting significantly declined from baseline to endline, likely due to secular improvements in socio‐economic status as well as the co‐location of multiple nutrition and health programmes.
- Repeat MNP coverage was independently associated with a reduced risk of anaemia and iron deficiency anaemia after adjustment for socio‐economic and programme‐related variables. The district with higher MNP coverage also experienced a significant reduction in the prevalence of anaemia, moderate/severe anaemia, and iron deficiency anaemia from baseline to endline.

## 1 INTRODUCTION

Undernutrition in early life—particularly from conception to 2 years of age—increases the risk of premature death and illness in childhood and also has lifelong consequences for child development (Black et al., 2013). Micronutrient deficiencies, often called “hidden hunger” because individuals with these conditions may not feel or appear hungry or deficient, are particularly prevalent globally—affecting over a third of people worldwide (Micronutrient Initiative, 2009). Among children aged 6–59 months in low‐ and middle‐income countries, 43% suffer from anaemia (Stevens et al., 2013), an estimated quarter of which is due to iron deficiency (Petry et al., 2016). Iron deficiency anaemia has been shown to impair child development, particularly the motor development of infants and young children, and also has negative consequences for cognition and schooling as children age (Black et al., 2013). Interventions designed to address multiple micronutrient deficiencies have gained particular attention because several deficiencies often cluster within the same individuals and communities (Allen, Peerson, & Olney, 2009; Christian & Tielsch, 2012; Neufeld & Ramakrishnan, 2011). Micronutrient powders (MNPs)—which come in single‐dose, light‐weight, shelf‐stable sachets—can be mixed with a variety of semi‐solid foods to increase the availability of vitamins and minerals in children's diets (Zlotkin et al., 2005). Meta‐analyses of randomized controlled trials have shown that regular MNP consumption can reduce the risk of anaemia in children aged 6–23 months by a quarter and can cut the prevalence of iron deficiency in half (De‐Regil, Suchdev, Vist, Walleser, & Peña‐Rosas, 2013; Salam, MacPhail, Das, & Bhutta, 2013); however, evidence from large‐scale programmes using MNPs is limited (Hirve et al., 2013; Jefferds et al., 2015; Menon et al., 2007; Nyhus Dhillon et al., 2017; Rah et al., 2012; Serdula et al., 2013; Suchdev et al., 2012; Vossenaar et al., 2017). In 2015, 65 countries implemented programmes using MNP reaching over 10 million children aged 6–59 months and providing them with MNP in 2015 (United Nations Children's Fund [UNICEF], 2017). Of the 65 countries implementing programmes with MNP in 2015, over three‐quarters have integrated MNP into infant and young child feeding (IYCF) programmes (UNICEF, 2017); however, there is limited evidence assessing the effectiveness of these integrated interventions, particularly when implemented at scale, on children's nutritional status.

Undernutrition is a major public health problem in Nepal where 36%, 10%, and 27% of children under 5 years are stunted, wasted, and underweight, respectively, and 46% of children 6–59 months suffer from anaemia (Nepal Demographic and Health Survey 2016: Key Indicators, 2017). The Ministry of Health, Nepal (MoH), in collaboration with the UNICEF, started developing an integrated IYCF–MNP programme targeting children aged 6–23 months in 2009 (Jefferds et al., 2015). After a feasibility study assessed the acceptability of MNP and developed key messages and strategies for implementing an integrated IYCF–MNP programme in Nepal, a pilot programme was implemented in 2010–2011 in six districts (Jefferds et al., 2015). In 2012, the MoH and UNICEF began scaling up the integrated IYCF–MNP programme, which as of 2016, had reached 25 of the country's 75 districts. In this study, we assess changes in the nutritional status (growth, anaemia, and iron deficiency) of children 6–23 months in two districts in Nepal that were part of a post‐pilot scale‐up of an integrated IYCF–MNP programme.

## 2 SUBJECTS AND METHODS

In Nepal, MNP has been locally branded as *Baal Vita, locally translated as* “vitamins for children.” Each sachet contains 15 micronutrients including 10 mg of elemental iron, 4.1 mg of zinc, 17 μg of selenium, 0.56 mg of copper, 90 μg of iodine, 400 μg of vitamin A, 5 μg of vitamin D3, 90 μg of folic acid, 6 mg of niacin, 0.9 μg of vitamin B12, 30 mg of vitamin C, and 0.5 mg of vitamins E, B1, B2, and B6 (WFP/WHO/UNICEF, 2007). The integrated IYCF–MNP intervention supports improved IYCF practices as well as the distribution of 60 sachets of MNP free of charge to all children aged 6–23 months every 6 months through local health facilities and through female community health volunteers (FCHVs). The programme used a cascade training approach with a manual based on the UNICEF (2012) community‐based IYCF training tools with additional information on the appropriate storage, distribution, and utilization of MNP adapted by the MoH for the Nepal context to train health workers and FCHVs. Health workers and FCHVs were in turn expected to counsel mothers during routine health visits and through community outreach. In accordance with the national FCHV strategy, FCHVs (who volunteer their time) were expected to hold monthly mothers group meetings where they would counsel mothers using the IYCF–MNP counselling cards, flipcharts, and cooking demonstrations and were also encouraged (but not required) to conduct home visits or individual counselling if they had the time to do so. FCHVs and health workers were also responsible for distributing 60 sachets of MNP to mothers every 6 months; mothers were instructed to feed their child one sachet per day, with an expected 4‐month gap after finishing the 60 sachets before the next distribution. The intervention also included updated counselling aids (e.g., IYCF flipcharts including MNP content, new posters, pamphlets, and radio jingles) re‐enforcing several key IYCF messages, with a focus on the timely introduction of complementary foods at 6 months of age; improving the energy density and nutritional quality of local complementary foods (including training on how to make home‐made superflour); information about MNP including the appropriate preparation and serving of food with MNP; and age appropriate minimum meal frequency, dietary diversity, and active feeding strategies. The IYCF content also emphasized early breastfeeding initiation, breast milk exclusivity for the first 6 months, and continued breastfeeding after 6 months of age for at least 2 years or longer, as well as sanitation messages related to safe water, handwashing, nail clipping, use of latrines, and appropriate disposal of child faeces.

The integrated IYCF–MNP programme held its first round of MNP distributions in Kapilvastu and Achham districts in March 2013; however, production issues originating with the international supplier resulted in a national stock‐out during the second half of 2013. There were no MNP distributions in the second half of 2013, and in the first half of 2014, the programme implemented a partial distribution with emergency MNP stock (generic MNP with the same micronutrient content as *Baal Vita* and a *Baal Vita* sticker on the box). IYCF counselling through mothers' groups and home visits continued throughout the MNP distribution delays, and the programme formally relaunched with refresher trainings of FCHVs and health facility staff and full MNP distributions in Kapilvastu and Achham in May and June 2014. The relaunched programme continued uninterrupted for over 18 months before the endline survey; thus, all children in the endline survey (who were aged 6–23 months) had been exposed to the full programme (without MNP stock‐outs) for 18 months or since they were eligible (as of age 6 months). This provided sufficient time for the oldest participants in the endline survey (children 18–23 months) to complete all three of their distribution cycles (60 MNP sachets every 6 months) after the relaunch but before the endline survey. Notably, in 2014, Suaahara, a multidistrict U.S. Agency for International Development‐funded programme, also initiated programme activities in Kapilvastu and Achham (Cunningham et al., 2017). Suaahara targeted mothers and children in the first 1,000 days with an integrated nutrition, health services, family planning, WASH, and agriculture/homestead food production programme. The Suaahara programme provided health workers and FCHVs with additional IYCF trainings; thus, many FCHVs and health workers received trainings from both the Suaahara and integrated IYCF–MNP programmes with overlapping IYCF messages (though Suaahara did not include an MNP component). Both programmes also supported IYCF key messages with mass media, and both programmes supported health worker and FCHV group meetings, home visits, and food demonstrations to strengthen IYCF practices among mothers of children under 23 months.

### 2.1 Survey design, sampling, and data collection

Prior to expanding to nine additional districts in 2013, a programme evaluation was designed for Achham and Kapilvastu districts (in the hills and plains ecological zones, respectively). Cross‐sectional baseline and endline surveys representative of children aged 6–23 months in the two districts were implemented prior to programme initiation in December to February 2012/2013 and then again in January to February 2016. During consultations on the evaluation design, the stakeholders of the integrated IYCF–MNP programme decided that it would not be practical to include “control” districts in the impact evaluation given the rapidly changing landscape of nutrition programmes in Nepal and the likelihood that additional nutrition interventions would be initiated in other districts between the baseline and endline surveys. The baseline survey sample size was calculated based on an estimated change in anaemia prevalence from 65% (Nepal Demographic and Health Survey, 2011) to 50% and iron deficiency from 50% to 40%. The results of the baseline survey data were used to estimate sample sizes for the endline survey. The assumed design effect for anaemia was 3.2 and 2.0 for iron deficiency. With an assumed household response rate of 95%, an individual response rate of 90%, and an overall response rate of 85.5%, we estimated the need for total sample size of 2,662 children 6 to 23 months in the two districts.

Both the baseline and endline surveys used a two‐stage cluster sampling method. Population proportion to size sampling selected 40 clusters from each district. A census in selected clusters identified all children aged 6–23 months. Using random sampling, at baseline, 32 children in Achham and 34 children in Kapilvastu were selected from each cluster; at endline, 34 children from each cluster were selected in Kapilvastu and 33 in Achham. There was no replacement for refusals or for clusters with less than the needed number of children. For each survey, field teams participated in 2 weeks of classroom and practical training including standardization exercises for length and weight and 3 days of pilot testing of all survey procedures in clusters in nearby districts not involved in the impact evaluation.

During both surveys, which were implemented at the household level, enumerators administered a questionnaire asking mothers about sociodemographic characteristics and IYCF knowledge and practices and also observed handwashing stations for presence of soap and water; at endline, mothers were also asked about programme exposure including details on the frequency and type of interactions with their FCHV, as well as their receipt and use of MNP. Mothers were also asked about MNP receipt and use at baseline, but no mothers reported MNP exposure at baseline. Data collection occurred during winter. For collection of length and weight, heavy outer clothing was removed leaving only lightweight clothing on the child. Children's length was measured to the nearest 0.1 cm using an Infant/Child/Adult ShorrBoard®. A Seca 874 Digital Floor Scale with Mother/Child Function to allow for the tarred weighing of young children held by the mother measured weight to the 0.01 kg. Trained laboratory technicians collected venous blood specimens from children and then assessed haemoglobin, malaria, and *Helicobacter pylori* (*H. pylori*) infections at the household using the HemoCue® Hb‐301 photometer (Angelholm, Sweden), the malaria antigen (HRP2/pLDH) combination rapid diagnostic kit for *Plasmodium falciparum* and *Plasmodium vivax*, and the *H. pylori* QuickVue rapid test kit, respectively. A disposable pipette transferred a drop of blood from the blood collection tube onto a piece of parafilm for assessing haemoglobin, malaria, and *H. pylori*. Although malaria is endemic in parts of Nepal, particularly during the hot season from April to October, at the time of our baseline and endline assessments (all from December to February), there was only one positive case of malaria at baseline and three at endline and no positive cases of *H. pylori*. The HemoCue® Hb‐301 photometer is self‐calibrating, but laboratory technicians also performed additional quality control procedures at the beginning of each day using three levels of liquid controls (low, normal, and high; Eurotrol, Burlington, MA, USA). The laboratory technicians placed the blood specimens into a cold box containing frozen gel packs and a thermometer. At a central portable laboratory established in the field in each cluster, the specimens were centrifuged on the same day (within 1–2 hr after collection), transferred into cryovials, and then stored in portable freezers. At the end of each day, the processed specimens were transferred to the freezers in the District Public Health Offices for storage until the end of data collection. At the end of the survey, all specimens from the District Public Health Offices were then transferred to the National Public Health Laboratory for storage at −86° centigrade. The specimens from National Public Health Laboratory were sent to the VitMin Laboratory (Willstaett, Germany) for the testing of ferritin, C‐reactive protein, and alpha‐1 acid glycoprotein presented in this analysis using an in‐house sandwich enzyme‐linked immunosorbent assay technique (Erhardt, Estes, Pfeiffer, Biesalski, & Craft, 2004).

Length‐for‐age, weight‐for‐length, and weight‐for‐age *z*‐scores (LAZ, WLZ, and WAZ) were calculated using the WHO (2006) growth standards ((WHO Multicentre Growth Reference Study Group & de Onis, 2006). Each child's age was back‐calculated by subtracting the child's birth date from the interview date. Stunting, wasting, and underweight were defined as <−2 standard deviations for LAZ, WLZ, and WAZ, respectively. In accordance with WHO recommendations, all extreme LAZ (<−6 or >6), WLZ (<−5 or >5), and WAZ (<−6 or >5) values were set to missing (WHO, 2006). As additional quality checks of length and weight data, we examined the per cent of missing data for age, sex, length, and weight; end‐digit preference for length and weight values; and *SD* for LAZ, WLZ, and WAZ. Each child's haemoglobin was adjusted for altitude based on the global positioning system coordinates of the household (WHO, 2011). Anaemia was defined as altitude‐adjusted haemoglobin <110 g/L. Moderate or severe anaemia was defined as altitude‐adjusted haemoglobin <100 g/L; children with moderate and severe anaemia were combined into a single category because only six and seven children in the baseline and endline surveys were classified as severely anaemic (haemoglobin <70 g/L). Serum ferritin was adjusted for inflammation (C‐reactive protein and alpha‐1‐acid glycoprotein) using the Biomarkers Reflecting Inflammation and Nutritional Determinants of Anemia linear regression technique with internal data‐driven reference levels (Suchdev et al., 2016). Iron deficiency was defined as inflammation‐adjusted serum ferritin <12 μg/L. Iron deficiency anaemia was defined as iron deficiency plus anaemia.

### 2.2 Ethical approval

Ethical approval was obtained from the Nepal Health Research Council for both the baseline and endline surveys. For each survey, interviewers described the purpose, procedures, risks, and benefits of the study and allowed mothers to ask questions before inviting mothers and children to participate in the survey. Mothers or other legal guardians then provided written informed consent to enrol themselves and their children in the study. If the mothers or guardians were illiterate, then a witness signature was obtained.

### 2.3 Statistical methods

Descriptive statistics—frequencies with proportions and means with standard deviations—were used to compare baseline and endline sociodemographic, nutrition, water, and sanitation and programme characteristics by district. Multivariable log‐binomial regression models were used to estimate the prevalence ratios for binary outcomes comparing children in the endline survey to those in the baseline survey (Spiegelman & Hertzmark, 2005), whereas continuous outcomes were compared using linear regression models; all models account for clustering using an exchangeable correlation structure. Covariates for multivariable models were selected a priori based on a review of the literature and include child's sex, age, caste, maternal education, household food insecurity level, and household asset tertile developed from a principal component analysis based on household ownership of electricity, radio, television, mobile, refrigerator, table, chair, bed, sofa, watch, computer, fan, traditional grain miller, and bicycle (Vyas & Kumaranayake, 2006). In order to explore the large reduction in stunting from baseline to endline in the two districts, the point and interval estimates of the per cent of the change from baseline to endline that was mediated by intermediate variables, such as sociodemographic characteristics, water, sanitation, hygiene, health, and IYCF indicators, were also estimated using log‐binomial regression models.

Because some programme exposure variables, such as MNP coverage and the frequency of maternal–FCHV interactions, were only available in the endline survey, log‐binomial models were used to assess the relationship between programme exposure variables and children's nutritional status in the endline survey only. In a previous analysis, we found that key IYCF practices (including feeding the child the minimum dietary diversity, meal frequency, and acceptable diet) improved in the programme area from baseline to endline and that several IYCF practices were significantly more prevalent among mothers who received IYCF counselling from an FCHV and interacted with their FCHV at least twice per month compared with mothers who did not receive FCHV IYCF counselling or interacted with the FCHV less than once per month (Locks et al., 2018). Because mother–FCHV interactions may have contributed to children's nutritional status through pathways external to discussing IYCF only (such as discussing WASH or child health more generally), in this analysis, we compare children's nutritional status solely based on the frequency with which their mothers interacted with their FCHVs for the child: frequent (>once per month) and intermediate (once per month) compared with infrequent interactions (<once per month). Because very few mothers reported “never” interacting with their FCHV, mothers who reported that they never interacted with their FCHV or did so less than once per month were collapsed into a single reference group. We also compared children's nutritional status based on the number of times the mothers received MNP for the child (≥2 MNP distributions or a single MNP distribution vs. no MNPs) among children 12–23 months (children <12 months were excluded because they were only expected to receive MNP one time). Cross‐sectional analyses from the endline survey adjust for the same sociodemographic characteristics above, as well as the location of maternal–FCHV interactions for the child: home visits, community meetings, or both (a proxy for maternal health‐seeking behaviour). In order to isolate the effect of MNP on children's nutritional status, models for MNP coverage also adjust for frequency of maternal–FCHV interactions for the child and whether the child received the minimum acceptable diet the previous day. The two districts were first modelled separately; however, because trends across the two districts were similar, the districts were combined, and district was added as a covariate. Because analyses assessing the relationship between programme indicators and health outcomes were not intended to provide population‐based estimates, sample weights were not applied to these analyses; all models account for clustering using an exchangeable correlation structure. All analyses were conducted in SAS 9.4 (Cary, NC, USA).

## 3 RESULTS

In all, 2,543 and 2,577 children participated in the baseline and endline surveys, respectively, reflecting response rates of 97% and 96% of targeted children (Table 1). In both the baseline and endline surveys in both districts, approximately half of the children were male, and one third fell into each of the age categories (6–11, 12–17, and 18–23 months). In the endline survey, 54.4% and 73.2% of mothers reported receiving MNP for their child at least once in Kapilvastu and Achham, respectively; no mothers had received or used MNP in the baseline survey. In both districts, over half of mothers who had received MNP reported receiving only one box (30 sachets) as opposed to the recommended two boxes (60 sachets) at their last distribution. Among children who consumed MNP, the mean number of sachets consumed from their last distribution (based on maternal report) was 24.7 in Kapilvastu and 23.6 sachets in Achham. Among mothers who never received MNP (*n* = 613 in Kapilvastu and 330 in Achham), 276 (45.0%) and 130 (39.4%) of mothers reported that they did not know they were supposed to get MNP for their child, and 113 (18.4%) and 67 (20.3%) reported a stock‐out at their FCHV/health facility in Kapilvastu and Achham, respectively (data not shown).

**Table 1:** Characteristics of study samples at baseline and endline in Kapilvastu and Achham districts, Nepal^a^

	Kapilvastu district		Achham district	
	2013	2016	2013	2016
	*n* = 1,286	*n* = 1,345	*n* = 1,257	*n* = 1,233
**Sociodemographic characteristics**				
Child is male	675 (52.5)	718 (53.4)	670 (53.3)	651 (52.8)
Child's age (months)^b^	14.6 ± 4.8	15.0 ± 5.0	15.0 ± 5.1	14.6 ± 5.0
Child's ethnicity				
Upper ethnic caste	187 (14.5)	189 (14.1)	827 (65.8)	840 (68.1)
Dalit	216 (16.8)	220 (16.4)	418 (33.3)	379 (30.8)
Other	883 (68.7)	936 (69.6)	12 (1.0)	14 (1.1)
Maternal education				
No formal education	751 (58.4)	623 (46.4)	863 (68.7)	663 (53.8)
Primary education	219 (17.0)	280 (20.8)	143 (11.4)	151 (12.3)
Secondary education or higher	316 (24.6)	441 (32.8)	251 (20.0)	418 (33.9)
Main source of household income				
Agriculture	811 (63.1)	769 (57.2)	998 (79.4)	737 (59.8)
Remittance	93 (7.2)	177 (13.2)	143 (11.4)	259 (21.0)
Other	382 (29.7)	399 (29.7)	116 (9.2)	237 (19.2)
Household food security^c^				
Food secure	658 (51.2)	908 (67.5)	458 (36.4)	486 (39.4)
Mildly food insecure	130 (10.1)	106 (7.9)	196 (15.6)	145 (11.8)
Moderately food insecure	466 (36.2)	207 (15.4)	440 (35.0)	248 (20.1)
Severely food insecure	32 (2.5)	124 (9.2)	163 (13.0)	354 (28.7)
Household asset tertile^d^				
Tertile 1 (more assets)	576 (44.8)	543 (40.4)	253 (20.1)	374 (30.3)
Tertile 2	396 (30.8)	607 (45.1)	261 (20.8)	379 (30.7)
Tertile 3 (fewer assets)	314 (24.4)	195 (14.5)	743 (59.1)	480 (38.9)
Water, sanitation, and health characteristics				
Source of drinking water				
Piped water	37 (2.9)	171 (12.7)	979 (77.9)	1,020 (82.7)
Tube well	1,228 (95.6)	1,153 (85.7)	0 (0.0)	2 (0.2)
Unimproved source	19 (1.5)	21 (1.6)	278 (22.1)	211 (17.1)
Type of toilet				
Flush toilet	362 (28.2)	597 (44.6)	740 (58.9)	631 (51.5)
Ventilated improved pit latrine	27 (2.1)	54 (4.0)	25 (2.0)	201 (16.4)
Traditional pit latrine	13 (1.0)	123 (9.2)	189 (15.0)	346 (28.2)
No toilet	884 (68.7)	564 (42.2)	303 (24.1)	47 (3.8)
Household participated in “Open Defecation Free” programme	200 (15.6)	302 (22.5)	440 (35.0)	486 (39.4)
Child has taken medication for deworming in past 6 months	681 (53.0)	745 (55.4)	857 (68.2)	840 (68.1)
Child has received vitamin A supplementation in past 6 months	1,103 (85.8)	1,010 (75.1)	1,144 (91.0)	1,009 (81.8)
**IYCF–MNP programme indicators**				
Mother reports child initiating solid foods at 6 months	432 (35.9)	834 (64.7)	603 (48.6)	998 (82.9)
Child was breastfed the previous day	1,210 (94.2)	1,249 (93.6)	1,219 (97.0)	1,189 (97.3)
Number of times the child was breastfed the previous day^b^	10.4 ± 4.7	10.1 ± 4.6	8.4 ± 4.0	9.6 ± 3.8
Child received the minimum dietary diversity the previous day^e^	289 (24.6)	547 (43.5)	321 (26.7)	423 (36.5)
Child received the minimum meal frequency the previous day^e^	527 (45.0)	744 (55.8)	692 (57.5)	686 (56.2)
Child received the minimum acceptable diet the previous day^e^	169 (14.5)	375 (30.1)	222 (18.5)	282 (24.6)
Child was fed home‐made superflour in the previous day	64 (5.0)	153 (11.4)	34 (2.7)	226 (18.3)
Household has a nail clipper	688 (53.5)	890 (66.2)	780 (62.1)	950 (77.1)
Household has a handwashing station with observed soap and water	799 (64.9)	1,004 (78.9)	363 (30.0)	547 (46.0)
Mother reports using soap in the previous or current day	1,148 (89.3)	1,298 (96.5)	809 (64.4)	1,138 (92.3)
Mother reports soap use after cleaning child's stool in the previous day	488 (38.0)	740 (55.0)	220 (17.5)	570 (46.2)
Mother reports soap use before preparing food/feeding child in previous day	105 (8.2)	236 (17.6)	30 (2.4)	214 (17.4)
Frequency of mother–FCHV interactions for the child				
<Once per month	—	523 (38.9)	—	315 (25.6)
Monthly	—	403 (30.0)	—	358 (29.0)
>Once per month	—	419 (31.2)	—	560 (45.2)
Location of mother–FCHV interactions	—			
Home visits	—	281 (23.6)	—	169 (14.6)
Elsewhere in the community	—	464 (39.0)	—	650 (56.3)
Both home visits and community visits	—	444 (37.3)	—	335 (29.0)
Mother ever received MNP for the child	—	732 (54.4)	—	903 (73.2)
Among mothers of infants 12–23 months^d^, mother received MNP ≥2 times	—	269 (20.0)	—	434 (35.3)
Among those who received MNP, received two boxes (60 sachets) at last distribution	—	299 (41.4)	—	382 (42.7)
Among those who received MNP, received one box (30 sachets) at last distribution	—	423 (58.6)	—	512 (57.3)
Child ever consumed MNP	—	719 (53.5)	—	880 (71.4)
Among MNP consumers, # sachets consumed by child from last batch received^b^	—	24.7 ± 20.0	—	23.6 ± 19.9

*Note*. IYCF–MNP: infant and young child feeding–micronutrient powder; FCHV: female community health volunteer.

a All values are frequency *n* (%) with *P* values from chi‐squared tests unless otherwise indicated.

b Values are mean ± standard deviation.

c Food security defined in accordance with the household food insecurity access scale (Coates, Swindale, & Bilinsky, 2007).

d Asset tertile from principal component analysis of list of radio, television, mobile phone, refrigerator, table, chair, bed, sofa, watch, computer, fan, traditional grain miller, bicycle, and electricity.

e Minimum dietary diversity, meal frequency, and acceptable diet (MDD, MMF, and MAD) defined based on the UNICEF/WHO IYCF indicators (WHO, 2008). MDD defined as ≥4 out of 7 food groups the previous day. MMF as solid or semi‐solid food ≥2× per day for breastfed infants 6–8 months, ≥3× for breastfed children 9–23 months, and ≥4× for nonbreastfed children 6–23 months. MAD for breastfed children is MMD and MMF in the previous day. MAD for nonbreastfed children defined as consumption in the previous day of ≥2 milk feeds, solid/semi‐solid food ≥4×, and ≥4 food groups (from a total of six groups that excludes dairy).

From the baseline survey 99.6% and 99.7% children had complete length and weight data, respectively; for the endline survey, 99.3% and 99.6% had complete length and weight data. There were no missing data for age or sex. In both districts, children in the endline survey were significantly less likely to be stunted than children in the baseline survey even after adjusting for key sociodemographic characteristics, source of household drinking water, type of toilet, and participation in community programmes including the Open Defecation Free, deworming, and vitamin A supplementation campaigns (adjusted prevalence ratio [APR; 95% CI]: 0.77 [0.69, 0.85], *P* < 0.001 and 0.82 [0.75, 0.91], *P* < 0.001 in Kapilvastu and Achham, respectively; Table 2). In multivariable models for Achham, children in the endline survey also had a significantly lower prevalence of underweight (APR [95% CI]: 0.82 [0.71, 0.93], *P* = 0.003), moderate or severe anaemia (APR [95% CI]: 0.67 [0.46, 0.99], *P* = 0.04), iron deficiency (APR [95% CI]: 0.85 [0.77, 0.94], *P* = 0.003), and iron deficiency anaemia (APR [95% CI]: 0.78 [0.64, 0.95], *P* = 0.01) compared with children in the baseline survey.

**Table 2:** Comparing nutritional status of children 6–23 months at endline versus baseline in Kapilvastu and Achham districts, Nepal

	Kapilvastu district				Achham district			
	2013 *n* = 1,286	2016 *n* = 1,345	Adjusted prevalence ratio or difference of means^a^^,^ b (95% CI)	*P*	2013 *n* = 1,257	2016 *n* = 1,233	Adjusted prevalence ratio or difference of means^a^^,^ b (95% CI)	*P*
Prevalence ratios								
Stunting^c^	582 (45.5)	444 (33.2)	0.77 (0.69, 0.85)	<0.001	677 (54.0)	508 (41.5)	0.82 (0.75, 0.91)	<0.001
Wasting^c^	181 (14.2)	174 (13.0)	1.05 (0.84, 1.30)	0.67	111 (8.9)	118 (9.7)	1.09 (0.82, 1.46)	0.53
Underweight^c^	423 (33.1)	374 (27.9)	0.94 (0.83, 1.06)	0.29	450 (35.9)	339 (27.6)	0.82 (0.71, 0.93)	0.003
Anemia^d^	597 (48.7)	600 (47.5)	0.98 (0.90, 1.07)	0.67	400 (32.6)	298 (30.0)	0.96 (0.83, 1.11)	0.58
Moderate or severe anemia^d^	197 (16.1)	212 (16.8)	1.13 (0.93, 1.38)	0.22	97 (7.9)	51 (5.1)	0.67 (0.46, 0.99)	0.04
Iron deficiency^e^^,^ f	639 (54.7)	709 (57.1)	1.01 (0.94, 1.09)	0.72	601 (51.2)	414 (43.2)	0.85 (0.77, 0.94)	0.003
Iron deficiency anemia^e^^,^ g	386 (33.1)	416 (33.5)	1.02 (0.90, 1.15)	0.76	281 (24.0)	169 (17.7)	0.78 (0.64, 0.95)	0.01
Difference of means								
Length‐for‐age *z*‐score	−1.91 ± 1.32	−1.46 ± 1.41	0.36 (0.23, 0.48)	<0.001	−2.05 ± 1.21	−1.68 ± 1.28	0.29 (0.17, 0.41)	<0.001
Weight‐for‐length *z*‐score	−0.83 ± 1.09	−0.86 ± 1.04	−0.11 (−0.21, −0.02)	0.02	−0.75 ± 0.94	−0.74 ± 0.97	−0.03 (−0.11, 0.05)	0.43
Weight‐for‐age *z*‐score	−1.59 ± 1.20	−1.39 ± 1.10	0.11 (−0.00, 0.22)	0.06	−1.61 ± 1.01	−1.42 ± 1.02	0.13 (0.04, 0.22)	0.003
Haemoglobin (g/L)^d^	109.4 ± 11.7	109.4 ± 11.6	−0.35 (−1.45, 0.75)	0.53	113.7 ± 10.4	114.4 ± 10.3	0.61 (−0.40, 1.62)	0.23
Serum ferritin (μg/L)^e^	16.6 ± 19.0	15.2 ± 17.0	−0.95 (−2.48, 0.57)	0.22	17.1 ± 19.9	17.5 ± 15.4	0.90 (−0.90, 2.70)	0.33

a Prevalence ratios and corresponding 95% confidence intervals and *P* values obtained from generalized estimating equations using a log link and binomial distribution, accounting for correlated errors within clusters using an exchangeable correlation structure. When the log‐binomial model did not converge (in the models for stunting), a Poisson distribution was used. For continuous variables, difference of means and corresponding 95% confidence interval and *P* values were obtained from generalized estimating equations using the identity link and normal distribution accounting for correlated errors within clusters using an exchangeable correlation structure.

b All models adjust for child's sex, age and caste, maternal education, household food insecurity level and asset tertile, source of drinking water, type of toilet, whether anyone in the household participated in the “Open Defecation Free” campaign, and whether the child received deworming medication and/or vitamin A supplementation in the previous 6 months.

c Stunting, wasting, and underweight defined as <−2SD for length‐for‐age, weight‐for‐length, and weight‐for‐age *z*‐scores, respectively (WHO, 2006).

d Adjusted for altitude using WHO guidelines (WHO, 2011); anaemia defined as altitude‐adjusted haemoglobin <110 g/L; moderate or severe anaemia defined as altitude‐adjusted haemoglobin <100 g/L. *N* for haemoglobin and anaemia is 1,226 in 2013 and 1,262 in 2016 for Kapilvastu and 1,226 and 995 for Achham in 2013 and 2016, respectively.

e Adjusted for inflammation using the Biomarkers Reflecting Inflammation and Nutritional Determinants of Anemia linear regression technique (Burke et al., 2017; Suchdev et al., 2016). *N* for serum ferritin is 1,169 in 2013 and 1,242 in 2016 for Kapilvastu and 1,173 and 958 for Achham in 2013 and 2016, respectively.

f Iron deficiency defined as inflammation‐adjusted serum ferritin <12 μg/L.

g Iron deficiency anaemia defined as inflammation‐adjusted serum ferritin <12 μg/L and altitude‐adjusted haemoglobin <110 g/L.

In analyses of the influence of demographic, health, and programme covariates on the observed changes in stunting from baseline to endline, we found that 43.2% (*P* = 0.002) and 36.8% (*P* = 0.01) of the change in the prevalence of stunting from baseline to endline in Kapilvastu and Achham, respectively, could be explained by sociodemographic characteristics and water, sanitation, health, and IYCF indicators that were measured in the baseline and endline surveys (Table 3). In the full multivariate model, where the IYCF and hygiene behavioural indicators were added to the model that already adjusted for sociodemographic characteristics and water, sanitation, and health indicators, the new addition of the IYCF and hygiene indicators explained an additional 30.5% (*P* = 0.02) and 16.3% (*P* = 0.19) of the change in the prevalence of stunting from baseline to endline in Kapilvastu and Achham, respectively.

**Table 3:** Explaining the change in the prevalence of stunting among children aged 6–23 months from baseline to endline in Kapilvastu and Achham, Nepal, using sociodemographic, water, sanitation, health, and IYCF–MNP programme indicators

	Kapilvastu				Achham					
	PR (95% CI)	Total % mediated by all covariates	*P* for mediation	% mediated by new covariates only	*P* for mediation	PR (95% CI)	Total % mediated by all covariates	*P* for mediation	% mediated by new covariates only	*P* for mediation
Univariate model for risk of stunting (length‐for‐age *z*‐score <−2; endline vs. baseline)	0.73 (0.66, 0.80)	—	—	—	—	0.77 (0.71, 0.84)	—	—	—	—
Model adjusted for sociodemographic characteristics only^a^	0.74 (0.67, 0.82)	5.4	0.23	5.4	0.23	0.82 (0.75, 0.89)	22.3	0.002	22.3	0.002
Model adjusted for sociodemographic + water, sanitation, and health indicators^b^	0.77 (0.69, 0.85)	18.2	0.03	13.6	0.02	0.82 (0.75, 0.91)	24.5	0.01	2.4	0.39
Model adjusted for sociodemographic + water, sanitation, and health indicators^b^ + IYCF and hygiene behaviours^c^	0.84 (0.74, 0.95)	43.2	0.002	30.5	0.02	0.85 (0.76, 0.95)	36.8	0.01	16.3	0.19

*Note*. IYCF–MNP: infant and young child feeding–micronutrient powder; PR: prevalence ratio.

a Sociodemographic variables include child's age, sex and caste, maternal education, household food insecurity level, and asset tertile.

b Water, sanitation, and health variables are indicators that were not targeted by the IYCF–MNP intervention. They include source of drinking water (piped water, tube well, or unimproved water source), type of toilet (flush toilet, improved pit latrine, unimproved pit latrine vs. no toilet), whether the household participated in the “Open Defecation Free” campaign, and whether the child was dewormed and/or received vitamin A in the previous 6 months.

c The IYCF and hygiene behaviours that were added last to the full multivariable model include all practice indicators that were targeted by the IYCF–MNP programme and were measured at baseline and endline. These include when the mother reports introducing complementary foods (before, after, or at 6 months), frequency of breastfeeding in the previous day, frequency of solid foods in the previous day, number of food groups consumed in the previous day, whether the mother engaged in any responsive feeding activity (singing, talking to or eye contact with the child while feeding) in the previous day, and whether the child consumed locally made superflour in the previous day. It also includes hygiene indicators that were targeted by the IYCF–MNP programme key messages including whether there was observed soap and water at the household handwashing station, whether the mother reports using soap the current or previous day, whether she reports washing her hands with soap before preparing food or feeding the child in the previous or current day, whether she reports using soap after cleaning the child's stool in the previous or current day, and whether the household has a nail clipper.

In multivariable analyses from endline, children of mothers who saw their FCHV at least twice per month had a significantly lower prevalence of stunting and underweight (APR [95% CI]: 0.87 [0.77, 0.99], *P* = 0.03 and 0.84 [0.72, 0.99], *P* = 0.04), and children of mothers who saw their FCHVs monthly had a lower prevalence of wasting (APR [95% CI]: 0.69 [0.50, 0.95], *P* = 0.02), compared with children whose mothers saw their FCHV less than once per month (Table 4). Among children 12–23 months, those whose mothers reported receiving at least two MNP distributions for the child had a significantly lower prevalence of anaemia (APR [95% CI]: 0.79 [0.65, 0.96], *P* = 0.02) and iron deficiency anaemia (APR [95% CI]: 0.75 [0.58, 0.95], *P* = 0.02) compared with children whose mothers never received MNP in models adjusted for sociodemographic characteristics, frequency and location of mother–FCHV interactions for the child, and whether the child received minimum acceptable diet in the previous day (Table 5).

**Table 4:** Comparing the nutritional status of children age 6–23 months in the endline survey based on frequency of maternal interactions with her female community health volunteer (FCHV) for the child, Nepal

	FCHV <1× per month (ref) *n* = 838	FCHV once per month *n* = 761			FCHV >once per month *n* = 979		
	*n* (%) or mean ± *SD*	*n* (%) or mean ± *SD*	Adjusted prevalence ratio or difference of means (95% CI)^a^^,^ b	*P*	*n* (%) or mean ± *SD*	Adjusted prevalence ratio or difference of means (95% CI)^a^^,^ b	*P*
Prevalence ratios							
Stunting^c^	323 (38.8)	294 (38.8)	0.95 (0.84, 1.08)	0.42	335 (34.5)	0.87 (0.77, 0.99)	0.03
Wasting^c^	108 (13.0)	69 (9.1)	0.69 (0.50, 0.95)	0.02	115 (11.9)	0.96 (0.75, 1.22)	0.76
Underweight^c^	256 (30.7)	214 (28.3)	0.93 (0.80, 1.08)	0.33	243 (24.9)	0.84 (0.72, 0.99)	0.04
Anaemia^d^	299 (41.5)	265 (39.0)	1.00 (0.89, 1.11)	0.93	334 (38.9)	1.04 (0.92, 1.18)	0.51
Moderate or severe anemia^d^	86 (11.9)	82 (12.1)	1.13 (0.88, 1.45)	0.33	95 (11.1)	1.17 (0.90, 1.52)	0.23
Iron deficiency^e^^,^ f	367 (51.8)	360 (54.0)	1.09 (0.99, 1.19)	0.08	396 (48.1)	0.96 (0.85, 1.09)	0.56
Iron deficiency anaemia^e^^,^ g	198 (28.1)	185 (27.7)	1.06 (0.92, 1.22)	0.44	202 (24.5)	0.98 (0.80, 1.20)	0.87
Difference of means							
Length‐for‐age *z*‐score	−1.63 ± 1.40	−1.60 ± 1.28	0.05 (−0.07, 0.18)	0.39	−1.48 ± 1.36	0.14 (0.02, 0.27)	0.02
Weight‐for‐length *z*‐score	−0.87 ± 1.06	−0.74 ± 0.98	0.10 (0.01, 0.20)	0.04	−0.80 ± 0.98	0.00 (−0.10, 0.10)	0.93
Weight‐for‐age *z*‐score	−1.49 ± 1.10	−1.37 ± 1.01	0.12 (0.01, 0.22)	0.03	−1.34 ± 1.06	0.10 (0.00, 0.19)	0.045
Haemoglobin (g/L)^c^^,^ d	111.3 ± 10.6	111.6 ± 11.4	−0.09 (−1.26, 1.07)	0.88	111.8 ± 11.8	−0.54 (−1.86, 0.79)	0.43
Serum ferritin (μg/L)^e^	16.0 ± 16.3	15.5 ± 16.1	−0.94 (−2.70, 0.83)	0.30	17.0 ± 16.6	0.03 (−1.82, 1.88)	0.97

a Prevalence ratios and corresponding 95% confidence intervals and *P* values obtained generalized estimating equations using a log link and binomial distribution, accounting for correlated errors within clusters using an exchangeable correlation structure. When the log‐binomial model did not converge (in the model for stunting), a Poisson distribution was used. For continuous variables, difference of means and corresponding 95% confidence interval and *P* values were obtained from generalized estimating equations with the identity link and normal distribution accounting for correlated errors within clusters using an exchangeable correlation structure. Because we did not intend to perform population‐based estimates, sample weights were not applied to the analysis.

b Adjusted models control for district, child's sex, age and caste, maternal education, household food insecurity level and asset tertile, and the location of mother–FCHV interactions for the child (home visits only, visits elsewhere in the community only, or both).

c Stunting, wasting, and underweight defined as <−2SD for length‐for‐age, weight‐for‐length, and weight‐for‐age *z*‐scores, respectively (WHO, 2006).

d Anaemia defined as haemoglobin <110 g/L. Moderate or severe anaemia defined as haemoglobin <100 g/L. Haemoglobin was adjusted for altitude (WHO, 2011). *N* for anaemia and haemoglobin is 720, 679, and 858 for FCHV <1× per month, FCHV once per month, and FCHV >once per month groups, respectively.

e Adjusted for inflammation using the Biomarkers Reflecting Inflammation and Nutritional Determinants of Anemia linear regression technique (Burke et al., 2017; Suchdev et al., 2016).

f Iron deficiency defined as serum ferritin <12 μg/L. *N* for serum ferritin and iron deficiency is 709, 667, and 824 for FCHV <1× per month, FCHV once per month, and FCHV >once per month groups, respectively.

g Iron deficiency anaemia defined as serum ferritin <12 μg/L and haemoglobin <110 g/L.

**Table 5:** Comparing nutritional status of children aged 12–23 months^a^ in the endline survey based on coverage of micronutrient powders (MNPs), Nepal

	No MNP *n* = 411	Received one MNP distribution *n* = 705			Received ≥2 MNP distributions *n* = 638		
	*n* (%) (reference)	*n* (%)	Adjusted prevalence ratio or difference of means^a^^,^ b (95% CI)	*P*	*n* (%)	Adjusted prevalence ratio or difference of means^a^^,^ b (95% CI)	*P*
Prevalence ratios							
Stunting^c^	174 (42.5)	315 (45.1)	1.05 (0.89, 1.23)	0.60	287 (45.2)	0.96 (0.83, 1.12)	0.63
Wasting^c^	58 (14.2)	86 (12.3)	0.89 (0.64, 1.25)	0.51	59 (9.3)	0.76 (0.53, 1.10)	0.15
Underweight^c^	137 (33.4)	226 (32.3)	1.00 (0.83, 1.20)	0.99	185 (29.1)	0.91 (0.75, 1.11)	0.37
Anaemia^d^	170 (46.1)	272 (44.3)	1.06 (0.95, 1.20)	0.29	170 (29.5)	0.79 (0.65, 0.96)	0.02
Moderate or severe anaemia^d^	61 (16.5)	80 (13.0)	1.05 (0.80, 1.38)	0.71	49 (8.5)	0.84 (0.57, 1.25)	0.39
Iron deficiency^e^^,^ f	221 (61.4)	346 (57.6)	1.01 (0.91, 1.12)	0.92	273 (48.7)	0.91 (0.81, 1.03)	0.13
Iron deficiency anaemia^e^^,^ g	128 (35.7)	190 (31.6)	1.03 (0.89, 1.19)	0.69	111 (19.8)	0.75 (0.58, 0.95)	0.02
Difference of means							
Length‐for‐age *z*‐score	−1.76 ± 1.30	−1.85 ± 1.25	−0.07 (−0.24, 0.09)	0.39	−1.79 ± 1.32	0.10 (−0.09, 0.28)	0.31
Weight‐for‐length *z*‐score	−0.92 ± 1.02	−0.86 ± 1.00	0.04 (−0.09, 0.18)	0.51	−0.79 ± 0.89	0.06 (−0.07, 0.20)	0.36
Weight‐for‐age *z*‐score	−1.54 ± 1.11	−1.54 ± 1.02	0.00 (−0.14, 0.14)	0.99	−1.47 ± 1.03	0.10 (−0.06, 0.25)	0.21
Haemoglobin (g/L)^c^^,^ d	110.0 ± 12.0	110.7 ± 11.7	−0.45 (−1.93, 1.03)	0.55	114.2 ± 11.4	1.77 (−0.02, 3.56)	0.05
Serum ferritin (μg/L)^e^	12.0 ± 10.8	13.5 ± 11.4	0.26 (−1.35, 1.87)	0.75	15.0 ± 12.8	1.01 (−0.57, 2.59)	0.21

a Analysis is restricted to children 12–23 months because children under 12 months were not expected to receive more than one MNP distribution.

b Prevalence ratios and corresponding 95% confidence intervals and *P* values obtained from log‐binomial regression models accounting for correlated errors within clusters using an exchangeable correlation structure. When the log‐binomial model did not converge (in the model for stunting), a Poisson distribution was used. For continuous variables, difference of means and corresponding 95% confidence interval and *P* values were obtained from linear regression models accounting for correlated errors within clusters using an exchangeable correlation structure. Because we did not intend to perform population‐based estimates, sample weights were not applied to the analysis. Multivariable models control for district, child's sex, age and caste, maternal education, household food insecurity level and asset tertile, mother–female community health volunteer interaction for the children frequency (<once per month, monthly, and >once per month) and location (home visits, visits elsewhere in the community, or both), and whether the child received the minimum acceptable diet in the previous day.

c Stunting, wasting, and underweight defined as <−2SD for length‐for‐age, weight‐for‐length, and weight‐for‐age *z*‐scores, respectively (WHO, 2006).

d Anaemia defined as haemoglobin <110 g/L, adjusted for altitude (WHO, 2011). Moderate or severe anaemia defined as haemoglobin <100 g/L, adjusted for altitude (WHO, 2011). *N* for anaemia and haemoglobin is 812, 808, and 633 for no MNP, one MNP distribution, and ≥2 distribution groups, respectively.

e Adjusted for inflammation using the Biomarkers Reflecting Inflammation and Nutritional Determinants of Anemia linear regression technique (Burke et al., 2017; Suchdev et al., 2016).

f Iron deficiency defined as serum ferritin <12 μg/L. *N* for iron deficiency and serum ferritin is 790, 791, and 615 for no MNP, one MNP distribution, and ≥2 distributions groups, respectively.

g Iron deficiency anaemia defined as serum ferritin <12 μg/L and haemoglobin <110 g/L.

## 4 DISCUSSION

In this analysis of baseline and endline surveys that were representative of children aged 6–23 months in two districts in Nepal that were part of the post‐pilot scale‐up of an integrated IYCF–MNP programme, we found that the prevalence of stunting declined substantially in both districts between the baseline and endline surveys. The prevalence of underweight, iron deficiency, iron deficiency anaemia, and moderate‐to‐severe anaemia also declined significantly in Achham district only, which is consistent with the greater programme coverage in Achham compared with Kapilvastu: 71.4% of children assessed at endline in Achham and 53.5% in Kapilvastu had consumed MNP and 45.2% of mothers in Achham reported seeing their FCHV for the child at least twice per month compared with 31.2% in Kapilvastu.

Our findings on the substantial reduction in the prevalence of stunting from baseline to endline are somewhat surprising; however, a recent study assessing Nepal Demographic and Health Survey data from 2001 to 2011 found that asset accumulation, health and nutrition interventions, maternal education gains, and improvements in sanitation have contributed to one of the fastest recorded national declines in child stunting worldwide (Headey & Hoddinott, 2015). National rates of stunting among children under 5 continued to fall in Nepal from 40.5% in 2011 to 35.8% in 2016 (Nepal Demographic and Health Survey, 2011; Nepal Demographic and Health Survey 2016: Key Indicators, 2017). In the Far Western region (where Achham is located) during the same period, the estimated prevalence fell from 46.4% to 35.9%, whereas the prevalence in the Western region (where Kapilvastu is located) did not change (37.4% to 37.5%). Our findings indicate a more rapid decline in the prevalence of stunting in Achham and Kapilvastu than Demographic and Health Survey estimates, the cause of which warrants further investigation. Randomized controlled trials of MNP have indicated that MNP alone is likely insufficient to improve anthropometry (De‐Regil et al., 2013); however, optimal IYCF practices—particularly complementary feeding of diverse, nutrient‐rich foods like animal‐source foods—have been shown to improve paediatric anthropometry (Dewey & Adu‐Afarwuah, 2008). We found that mothers who interacted with an FCHV at least twice per month were significantly less likely to have children who were stunted or underweight. These findings are consistent with a previous analysis from this programme that showed that several key IYCF practices (including minimum dietary diversity and minimum acceptable diet) improved significantly from baseline to endline in both districts and that mothers who received IYCF counselling from an FCHV and interacted with their FCHV at least twice per month were significantly more likely to engage in several key IYCF practices (Locks et al., 2018). Taken together, the evidence indicates that the co‐location of the integrated IYCF–MNP programme and the *Suaahara* programmes, both of which substantially invested in strengthening the capacities of FCHVs in child nutrition and IYCF, combined with secular trends in underlying socio‐economic status, likely explains the observed improvements in child stunting and underweight. Notably in our analysis, mothers who saw their FCHV monthly were significantly less likely to have a child who was wasted compared with mothers who saw their FCHVs less than once per month, but frequent (>once per month) interactions were not associated with a reduced prevalence of wasting. It is possible that in this cross‐sectional analysis, there is reverse causation and that mothers of children who recently became wasted also recently began visiting their FCHV more frequently than the average monthly interaction.

In mediation analyses, we were able to attribute 43.2% and 36.8% of the decline in the prevalence of stunting to changes in sociodemographic characteristics and water, sanitation, hygiene (WASH), health, and IYCF indicators that were measured in our baseline and endline surveys. When the IYCF and hygiene behaviours (that were targeted by both the integrated IYCF–MNP and Suaahara programmes) were added to the multivariable model that already contained sociodemographic, water, sanitation, and health indicators, we found that the IYCF and hygiene indicators explained an additional 30.5% (*P* = 0.02) and 16.3% (*P* = 0.19) of the reduction in stunting prevalence in Kapilvastu and Achham, respectively. These findings indicate that improvements in handwashing and IYCF practices, which were supported by both the integrated IYCF–MNP and Suaahara programmes through FCHVs, may have contributed to some but not all of the observed reductions in stunting. Notably, in the mediation analysis in Achham, changes in sociodemographic indicators explained the majority of the reduction in stunting.

The finding that mother–FCHV interactions at least twice per month was associated with a reduced prevalence of underweight and stunting and also our previous finding that frequent FCHV interactions with FCHV–IYCF counselling were associated with select IYCF practices (Locks et al., 2018) warrant further research. Due to the burden on FCHVs, who volunteer their time and in some cases need to travel long distances between houses, it may not be feasible to design IYCF–MNP programmes that routinely include contact with FCHVs or other community health workers more than once a month. Implementation research examining FCHV–mother interactions and what leads to greater frequency of contacts for the child may help explain why these children had better growth outcomes and can inform programme design (Reerink et al., 2017).

We also found that the prevalence of iron deficiency, iron deficiency anaemia, and moderate–severe anaemia reduced significantly in Achham district from baseline to endline. Furthermore, in the endline analysis, repeat MNP coverage was independently, positively associated with children's haemoglobin level and a reduced risk of anaemia and iron deficiency anaemia, even after adjustment for sociodemographic characteristics, whether the child received the minimum acceptable diet the previous day, the frequency of mother–FCHV interactions, and maternal health seeking (whether she met the FCHV during home visits only, elsewhere in the community only, or both). Meta‐analyses of randomized trials have indicated that regular use of MNP can reduce the risk of anaemia and iron deficiency in children 6–23 months by a quarter and a half, respectively (De‐Regil et al., 2013; WHO, 2016). As is often the case in large‐scale programmes outside of a clinical setting, we found that the micronutrient status of children in Achham district improved to a lesser degree than what was achieved in randomized trials. However notably, our findings demonstrate that integrated IYCF–MNP programmes that are part of national and subnational programmes implemented at scale can still contribute to reductions in anaemia and iron deficiency even in the face of implementation barriers. The limited programme impact was likely due to implementation barriers including MNP stock‐outs at the FCHV and health facility level and lack of awareness among some mothers about MNP. In addition, the majority of mothers who received MNP reported receiving only one box of MNP (30 sachets) at the last distribution as opposed to the recommended two boxes. In the end, 71.4% and 53.5% of children 6–23 months had consumed MNP in Achham and Kapilvastu, respectively, and among those who tried it, the mean number of sachets consumed from the last distribution was less than one box of 30 sachets. Implementation research on how to maximize MNP coverage (Jefferds et al., 2015) and intake adherence (Mirkovic et al., 2016) will be essential for improving future programmes using MNP (Tumilowicz, Schnefke, Neufeld, & Pelto, 2017).

Limitations of our study include the cross‐sectional nature of the baseline and endline surveys and the lack of comparison group; it is thus possible that some of the observed changes in nutritional status between baseline and endline are due to secular trends not related to the IYCF–MNP programme. In the endline analyses assessing the association of frequency of mother–FCHV interactions and MNP coverage with children's nutritional status, we are also limited by the lack of randomization of the exposures, and thus, underlying differences between mothers who saw their FCHV frequently versus infrequently, or among children who received MNP versus those who did not, may ultimately be confounding the associations between programme exposure and nutrition indicators. We did, however, adjust for several demographic covariates, including maternal literacy and socio‐economic variables, which did not substantially change our findings. Last, some data, including IYCF practices and experiences with FCHV, are maternal report and subject to various biases including socially desirability and time recall; though notably in the Nepal pilot IYCF–MNP programme, maternal recall of the number of sachets consumed and the directly observed difference (based on subtracting the number of observed unopened sachets from the number received) were highly correlated (Ng'eno et al., 2017).

## 5 CONCLUSION

In two districts in Nepal that were part of a post‐pilot scale‐up of an integrated IYCF–MNP programme, the prevalence of stunting significantly declined from baseline to endline in both districts, likely due to secular improvements in socio‐economic status as well as the co‐location of multiple nutrition and health programmes. Repeat MNP coverage was independently associated with a reduced risk of anaemia and iron deficiency anaemia after adjustment for socio‐economic and programme‐related variables. Achham, the district with higher MNP coverage, also experienced a significant reduction in the prevalence of anaemia, moderate/severe anaemia, and iron deficiency anaemia from baseline to endline. As Nepal, and other national programmes, continue to scale‐up integrated IYCF–MNP programmes, embedded implementation research on how to achieve and sustain high MNP coverage and adherence will be essential for maximizing the benefit of integrated IYCF–MNP programmes.

## CONFLICTS OF INTEREST

The authors declare that they have no conflicts of interest.

## AUTHOR CONTRIBUTIONS

MEJ, PD, ZM, DW designed the evaluation; PD, RP, SC, NP, NJ and PL oversaw program implementation; PD, RP, SC, NP, NJ, PL, MEJ, ZM, and DW provided training, supervision and support of data collectors, and provided interpretation and approval of analyses; LML analyzed data; and LML, AG, MEJ wrote the paper and had primary responsibility for final content. All authors read and approved the final.
